# Posterior reversible enzephalopathie syndrome (PRES) following vestibular schwannoma surgery – Case report and review of the current theories on pathophysiology of PRES

**DOI:** 10.1016/j.bas.2024.104167

**Published:** 2024-12-24

**Authors:** Solveig Stadsholt, Aivars Strauss, Jenny Kintzel, Stefan Schob, Erck Elolf, Mareike Rutenkröger, Christian Strauss, Christian Scheller, Sandra Leisz, Julian Prell, Maximilian Scheer

**Affiliations:** a Department of Neurosurgery, University Hospital Halle, Ernst-Grube-Straße 40, 06120, Halle, Germany; b Department of Radiology, University Hospital Halle, Ernst-Grube-Straße 40, 06120, Halle, Germany; c Department of Medical Psychology, University Medical Center Hamburg-Eppendorf, Martinistraße 52, 20246, Hamburg, Germany

**Keywords:** Posterior reversible enzephalopathie syndrome, Vestibular schwannoma, Acoustic neuroma, Metronidazole, Encephalopathy

## Abstract

**Introduction:**

Posterior reversible encephalopathy syndrome (PRES) is an acute form of encephalopathy. Main characteristic of this syndrome is the development of subcortical/cortical edema in the occipital lobes. The most common causes are diseases such as pre-eclampsia, autoimmune diseases, allogeneic stem cell transplantation and after treatment with immunosuppressants or cytostatics. However, PRES is also occasionally observed in connection with neurosurgical procedures, particularly in the posterior fossa in pediatric patients.

**Research question:**

PRES in adults is extremely rare. After cranial surgery, the impaired consciousness caused by this syndrome may be misdiagnosed.

**Material and methods:**

We present a rare case of PRES associated with vestibular schwannoma (VS) surgery and metronidazole use and have conducted a literature review.

**Results:**

We found only two cases of PRES after surgery of a VS in the literature and three cases in connection with the administration of metronidazole. All cases involved women but the onset of symptoms was highly variable. The constellation of surgery and administration of metronidazole has not yet been described.

**Discussion and conclusion:**

The purpose of this review is to raise awareness of a very rare complication such as PRES in this setting. Antibiotics should be chosen carefully after such an operation, as this syndrome can be triggered by certain substances.

## Highlights

• First Case Report of PRES after surgery of a VS in combination with the administration of Metronidazole.
• Literature Review of all cases of PRES after VS surgery and in connection with metronidazole.
• Review includes both theories of pathophysiological background.

## 1 Introduction

The Posterior Reversible Encephalopathy Syndrome (PRES) is an acute form of encephalopathy characterized by the development of subcortical/cortical edema in the posterior part of the cerebral hemispheres. It is most commonly associated with conditions such as preeclampsia, autoimmune diseases, allogeneic stem cell transplantation, and post-treatment with immunosuppressants or cytostatics (Geocadin, 2023; Triplett et al., 2022). However, it also frequently occurs after the resection of intra-axial brain tumors and posterior fossa tumors in pediatric patients (González Quarante et al., 2016). There are many theories on PRES pathophysiology suggested to date, oftentimes controversial and contradictory, without any existent unified system.

The syndrome has been described in all age groups with a mean age of 45 years, with a female predominance even after preeclampsy/eclampsy patients excluded. There is no good data depicting prevalence so far, probably due to vague diagnostic criteria, small case series and sometimes confusing terminology.

Clinically, there is a rapid onset of qualitative and quantitative loss of consciousness, epileptic seizures, headache, and cortical visual disturbances. When diagnosed, there is often severe hypertension (Bartynski, 2008a).

However, it should be emphasized that the constellation of impaired consciousness and hypertension following surgical treatment of a posterior fossa finding can have many and more common causes, such as hydrocephalus or postoperative bleeding (Dubey et al., 2009).

A CT scan and/or MRI confirm the diagnosis. Treatment consists of symptomatic care and avoidance of possible triggering factors (i.e. hypertension, renal failure, immunosuppressants ect.), in absence of specific therapeutic options to date.

A condition that can have a similar presentation is reversible cerebral vasoconstriction syndrome (RCVS) (Erhart et al., 2023). Clinically, patients often present with thunderclap headache with or without focal neurological deficits (Singhal, 2023). Similar to PRES, edema or haemorrhages may be seen on imaging (Perillo et al., 2022). RCVS is also female-dominated and typically associated with elevated blood pressure (Cossu et al., 2019). Post-partum women appear to be particularly at risk of both diseases (Cossu et al., 2019). Although the pathophysiology of these diseases is not fully understood, it is likely that they involve transient blood flow dysregulation and endothelial dysfunction (Motolese et al., 2023). Because of the overlap in clinical presentation, neuroradiological and pathophysiological features, some authors do not consider the two syndromes as distinct entities but rather as a continuum of disease (Motolese et al., 2023; Pilato et al., 2020; Chen et al., 2020; Jeanneret et al., 2022).

In this case report, we present a rare case of PRES after resection of a vestibular schwannoma (VS). Since it is neither common nor easy to identify, we believe it is important to collect these rare cases and performed a review of the literature, in order to gain new insights through retrospective analysis.

## 2 Case report

**Fig. 1:**
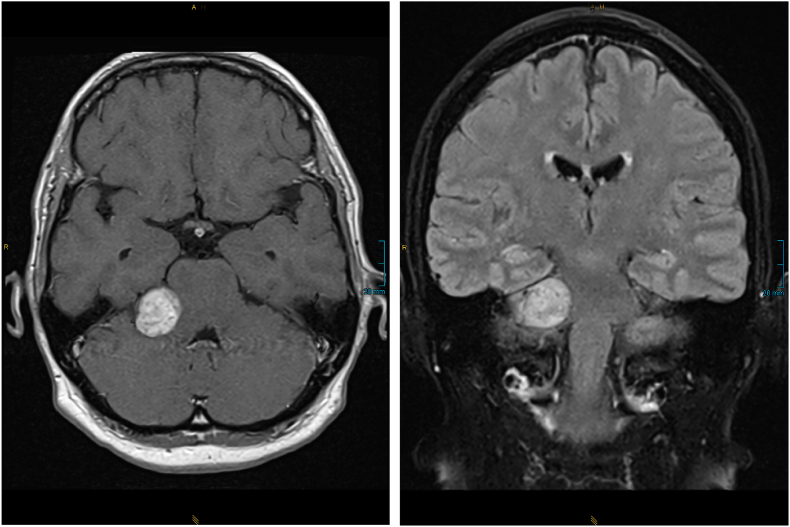
Preoperative MRI: axial T1weighted image post contrast and coronal FLAIR weighted image showing the tumor in the cerebellopontine angle.

A 52-year-old woman presented to our department for surgical treatment of a large VS (Koos grade 4) on the right side (Fig. 1). She complained of pressure in the right ear, dizziness and nausea for three weeks. On examination, she presented with right-sided hypoacusis, right-sided facial hypoesthesia and left-sided deviation on the Unterberger step test.

During tumour resection, the vestibulocochlear nerve could not be preserved. Before the anesthesia induction the patient was mildly hypertonic (150/80 for about 15 min), intraoperatively arterial pressure was in the range 110–130 systolic and 50–70 diastolic, and became briefly mildly hypertonic prior to extubation (maximal arterial pressure of 160/90). In postoperative ward systolic pressure stayed in the range 130–150 and diastolic 50–70. In the first two postoperative days the mean systolic pressure was 131 briefly rising to 158 and 159 on two occasions, and mean diastolic pressure was 62 with a maximum value of 88 on one occasion. All in all, there were no serious hypertensive events intraoperatively and in the early postoperative course. Intraoperatively, the patient received standard antibiotic prophylaxis with 2 g of cefazolin, which was repeated after 4 h (total duration of surgery 6 h). She had not taken any antibiotics in the previous months.

Postoperatively, the patient developed right-sided facial paresis (House Brackmann grade IV), but reported minimal to no headache, nausea or dizziness.

On post-operative day 2, the patient developed watery diarrhea, which was caused by Clostridium difficile. The moderate diarrhea was compensated by administration of jonosterile and did not lead to an increase in heart rate or laboratory evidence of hypovolemia. Oral Treatment with 1200 mg metronidazole per day was started in accordance with guidelines and resulted in rapid improvement of the gastrointestinal symptoms. Due to the disorder of consciousness, the oral administration was changed to an intravenous administration after two days.

**Fig. 2:**
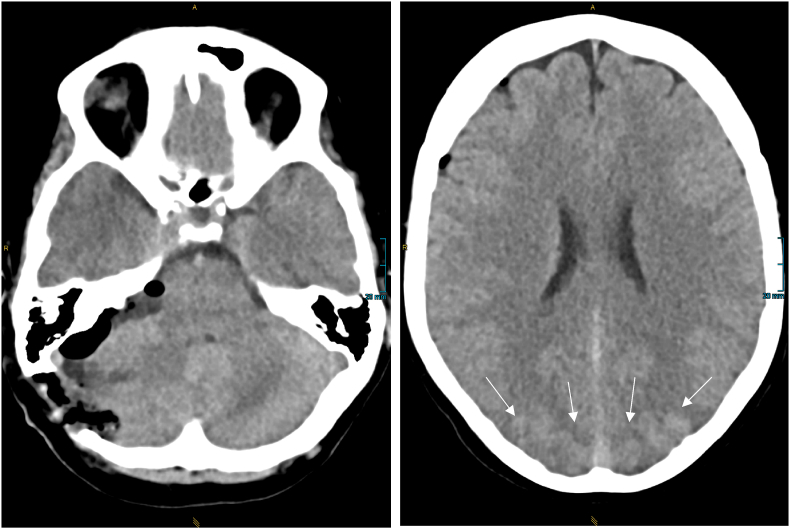
Non enhanced cranial computed tomography post resection showing subtle hypodensity of both occipital poles (marked with asterisks) as a first sign of PRES.

Progressive headache, dizziness and nausea developed during the third postoperative day. A cranial CT scan (first after surgical treatment) showed normal postsurgical findings without further pathologies. After the symptoms continued to worsen, another CT scan performed in the morning of the following day and ruled out intracranial hemorrhage or air insufflation but showed subtle hypodensity of both occipital poles as a first sign of PRES (Fig. 2).

**Fig. 3:**
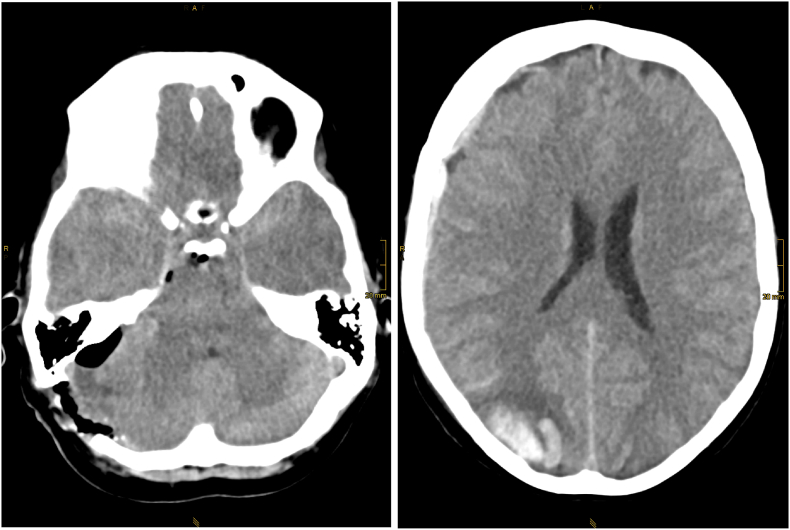
Follow up CCT 24h later – significant hemorrhage at the site of both occipital poles with a larger hematoma at the right hand side.

On the following day, the patient then presented with progressive, severe headaches and new quantitative and qualitative disturbances of consciousness. The main symptom was somnolence from the fourth postoperative day onwards, which lasted for three days. Repeated CT demonstrated a slightly progressive right parieto-occipital diffuse cerebral swelling, a right-sided intracerebral hematoma and a subdural hematoma with bilateral components of subarachnoid hemorrhage (Fig. 3). The patient was transferred to the intensive care unit (ICU). While no definite ictal events occurred, she developed left abducens paresis and left hemianopsia, as well as hypertension (maximum systolic blood pressure 160 mmHg).

**Fig. 4:**
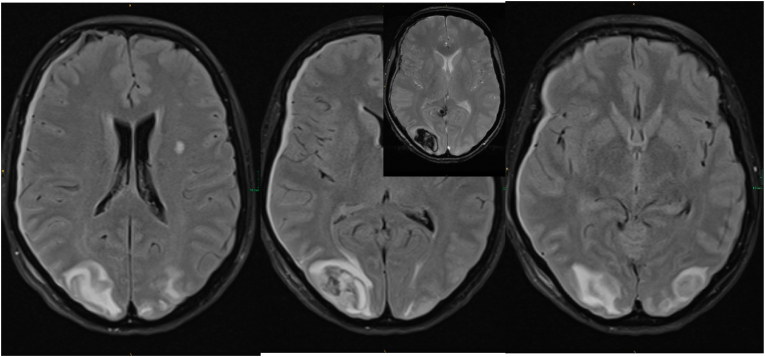
Follow up MRI (three subsequent FLAIR weighted images from cranial to caudal with a corresponding T2∗ section in the upper left corner of the middle image) again 24h later showing both, significant white matter hyperintensity reflecting vasogenic edema and subacute hematoma.

Based on the radiological and clinical signs, the diagnosis of PRES was considered. A cranial MRI performed on the fifth postoperative day showed diffuse subcortical T2 signal enhancement also in the area of the border zones frontal to parietal bilaterally, which further supported the diagnosis of PRES (Fig. 4). In addition, pressure-related diffusion abnormalities were observed in the right cerebellum, possibly affecting the abducens nerve due to depletion of the prepontine basal cisterns.

For neuroprotection, nimodipine was administered intravenously. In addition, urapidil was administered to treat arterial hypertension. Four days after first suspicion of PRES and possible correlation with the administration of metronidazole, the antibiotics were changed to oral vancomycin. The patient's condition improved rapidly. The patient was then transferred to the Intermediate Care Unit after three days and later to the general ward.

**Fig. 5:**
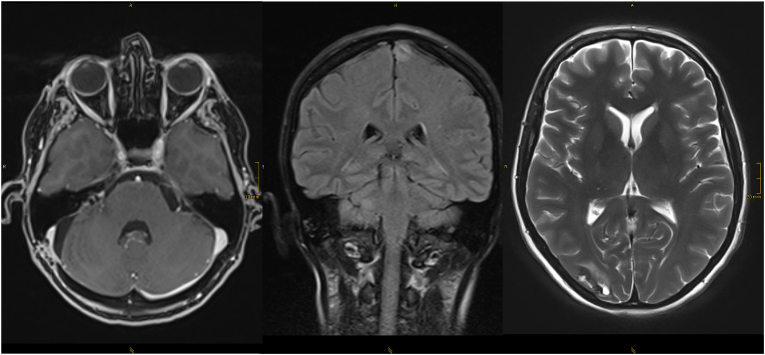
Follow up MRI (T1 Vibe post contrast axial reconstruction, coronal FLAIR, axial T2w section, from left to right) two months after resection of the tumor showing complete resolution of the acute PRES with hemosiderin residues visible at the right hand side occipital pole in the T2w image.

**Fig. 6:**
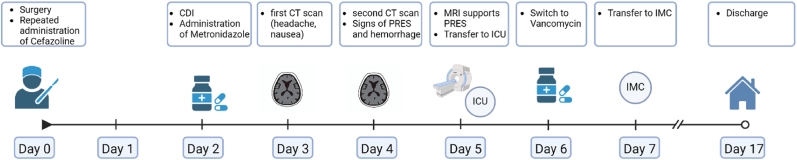
Timeline of the case report.

On discharge, 17 days after surgery, the patient had unchanged right-sided facial paresis (grade IV), no headaches, mild dizziness and a slight tendency to lean to the right when walking. The patient's oral antihypertensive medication was adjusted to amlodipine 5 mg once daily and ramipril 2.5 mg twice daily, which effectively maintained regular blood pressure. A follow-up MRI two months after the event showed complete regression of the PRES syndrome with residual haemosiderin in the area of the hemorrhage (Fig. 5). A timeline of this case report can be seen in Fig. 6.

## 3 Literature review

We conducted a systematic literature search on this topic using the PubMed, LIVIVO and Google Scholar using the following search terms: “Posterior reversible encephalopathy syndrome” AND “vestibular schwannoma”; “PRES” AND “vestibular schwannoma”; “Posterior reversible encephalopathy syndrome” AND “acoustic neuroma”; “PRES” AND “acoustic neuroma”; “Posterior reversible encephalopathy syndrome” AND “metronidazole”; “PRES” AND “metronidazole”. We found two case reports of PRES after VS surgery in adults (Table 1) and three case reports of PRES associated with metronidazole (Table 2).

**Table 1:** Characteristics of the case reports of PRES after surgery of a VS.

	Tumor	Approach	Age	Gender	Onset
Case 1	Vestibular schwannoma, Koos 4	retrosigmoid	23	Female	8 months
Case 2	Vestibular schwannoma, Koos 4	retrosigmoid	57	Female	2 days

**Table 2:** Characteristics of the case reports of PRES associated with metronidazole.

	Diagnosis	Treatment	Age	Gender	Onset
Case 1	Diveriticulitis	Metronidazole, Ciprofloxacin	75	Female	3 weeks
Case 2	Liver abscess	Metronidazole, evofloxacin	87	Female	2 weeks
Case 3	Ulcerative colitis	Metronidazole	25	Female	1 day

Both cases of PRES following surgical treatment of VS were women with a large tumor (Koos grade 4) treated surgically via a retrosigmoid approach. In case 1, the patient was 23 years old and the PRES did not occur until 8 months after the surgery and was associated with tumour progression (Khatri et al., 2018). In the second case, the patient was 57 years old and the symptoms were already observed on the second postoperative day (Sorour et al., 2016). In both cases, the typical radiological signs were accompanied by seizures and arterial hypertension.

The three case reports of PRES in connection with the use of metronidazole were also exclusively women. In case 1, the patient was 75 years old and was receiving metronidazole and ciprofloxacin due to diverticulitis. The onset of symptoms of PRES was observed approximately three weeks after the start of therapy (Gilbert et al., 2020). In case 2, the patient was 87 years old and was receiving metronidazole and levofloxacin as oral therapy for a previously intravenously treated liver abscess. She developed PRES two weeks after starting oral therapy (Hosaka et al., 2023). In the last case, the patient was 25 years old and received metronidazole due to ulcerative colitis. She developed symptoms of PRES within the first day (Kikuchi et al., 2016).

The main diagnostic tool for suspected PRES is a cranial MRI, which usually shows symmetrical bilateral occipital sub-/cortical edema. However, less commonly, it may be parietal, temporal, unilateral, or extend to the cerebellum and brainstem (Fugate and Rabinstein, 2015). In a study by Kastrup et al., 50 patient scans with PRES were retrospectively examined. These showed significantly more frontal involvement in FLAIR and DWI than previously assumed. Reversibility was independent of DWI pathology (Kastrup et al., 2015).

PRES per se has a good short- and long-term prognosis (Geocadin, 2023; Fischer and Schmutzhard, 2017). According to a follow-up study by Roth et al., the symptoms of PRES patients disappeared after an average of 7.5 days. Resolution of MRI lesions occurs more slowly than clinical recovery. The radiological follow-up examinations were unremarkable in almost a quarter of the patients. Two patients out of 25 suffered a reccurence of PRES (Roth and Ferbert, 2010).

Vertebrobasilar ischemia, cerebral vasculitis and encephalitis (autoimmune, viral) are seen as important differential diagnoses. The therapy currently consists of antihypertensive and anticonvulsant therapy. In case of suspected triggers for the condition, their elimation should be the goal – as far as possible (Bartynski, 2008a, 2008b; Bartynski, 2008a; Anderson et al., 2020; Bartynski, 2008b). Bleeding occurs occasionally, although secondary hemorrhage is often suspected. To date, no pathological alterations specific to PRES have been detected in cerebrospinal fluid (CSF). Elevated CSF protein levels correlate with the extent of cerebral edema (Datar et al., 2015a).

The pathophysiology of PRES has not yet been sufficiently researched and is largely controversial. Most authors agree that it is multifactorial in origin and that different mechanisms may predominate in subgroups of patients (Feske, 2011). The initial theory is that of reduced perfusion, but in recent years, the theory of hypertension/hyperperfusion has gained support (Servillo et al., 2007; Striano et al., 2005; Kwon et al., 2001). There is also a general agreement that endothelial injury/dysfunction is part of the pathogenesis (Fugate and Rabinstein, 2015; Marra et al., 2014).

### 3.1 Hypertension/hyperperfusion theory

It is well observed that most patients with PRES are hypertensive (Bartynski, 2008a). There is also a strong evidence that the blood flow in the brain is subject to autoregulation and is kept largely independent of the mean arterial pressure (MAP). Here, the so-called neurovascular unit (endothelium, glia and neurons) is of crucial importance (Schaeffer and Iadecola, 2021), as well as a number of cytokines (Marra et al., 2014). A generally accepted range where autoregulation takes place extends from 50 to 150 mmHg, but is subject to several influencing factors (Hall and Hall, 2021; GREENFIELD's NEUROPATHOLOGY 10E VOL 2, 2023). For instance, in patients suffering from chronic arterial hypertension, the upper MAP limit is considered to be above 150 mmHg (Hall and Hall, 2021). When MAP (which is usually around 90 mmHg in a healthy person at rest) exceeds the upper limit of autoregulation, vasogenic edema may develop (Auer, 1978; Kontos et al., 1978; MacKenzie et al., 1976). It has not been conclusively clarified whether arterial hypertension plays a triggering or accompanying role in the development of PRES (Bartynski, 2008a; Fugate and Rabinstein, 2015; Marra et al., 2014). Fluid leakage from the capillary bed in case of extreme arterial hypertension with the resulting hyperperfusion and disruption of the blood-brain barrier has been demonstrated in animal experiments with induced hypertension. In humans, the same result was obtained using single photon emission computed tomography (SPECT) with Technetium-99m hexamethyl propylenamine oxime (Tc-99m-HMPAO) (Schwartz et al., 1995; Apollon et al., 2000; Lewis et al., 1992; Rabinstein et al., 2012; Sudulagunta et al., 2017). This hypothesis might explain the cases of patients with malignant arterial hypertension (including those of secondary hypertension, such as a result of a pheochromocytoma, or renal hypertension), pre-eclampsia/eclampsia patients, as well as cases of medication/drug-induced hypertension.

Vasogenic edema that occurs when the hypertensive situation exceeds the limits of autoregulation has been demonstrated in animal experiments (MacKenzie et al., 1976). According to the authors of this study, this theory is supported by the fact that PRES lesions are predominantly reversible if antihypertensive therapy is started quickly. In addition, the predominantly affected area supplied by the posterior circulation, is predestined for hypertensive vasogenic edema due to the lack of sympathetic tone (Fugate and Rabinstein, 2015). in contrast to the anterior circulation, which is more densely innervated by the superior cervical ganglion. The fact that the extent of cerebral edema does not correlate with the severity of hypertension speaks against the hypothesis (Bartynski, 2008a, 2008b; Bartynski, 2008a; Bartynski, 2008b). In addition, there is evidence that up to 30% of those affected by PRES are normotensive or only mildly hypertensive (Bartynski, 2008a, 2008b; Bartynski, 2008a; Bartynski, 2008b; Bartynski et al., 2001; Bartynski and Boardman, 2008). Furthermore, MAP exceeds the above-mentioned autoregulation limit in less than 50% of patients (Li et al., 2012). In addition, several studies using SPECT and MR perfusion have shown low-perfusion areas of the brain in PRES-patients (Bartynski and Boardman, 2008; Naidu et al., 1997; Brubaker et al., 2005; Engelter et al., 1999).

More recent studies focus on endothelial dysfunction as a central factor in the pathogenesis, distin-guishing between “cytotoxic” and “immunogenic” causes (Hinduja, 2020). The cytotoxic theory describes primary vascular damage caused by endogenous cytokine release as in sepsis or exogenous toxins such as cytostatics, calcineurin inhibitors and immunosuppressants; in patients with such conditions, PRES has been frequently observed (Hinduja, 2020). The immunogenic thesis assumes T-cell activation and excessive cytokine release (Tumor Necrosis Factor (TNF)-α, Interleukin (IL)-1, Interferon-γ and IL-6). Endothelial surface antigens (P-selectin, E-selectin, intercellular adhesion molecule (ICAM) 1, vascular cell adhesion protein (VCAM)-1) are upregulated, resulting in increased leukocyte adherence and impaired microcirculation, whereupon autoregulation is disturbed. Edema formation may be the consequence of this (Marra et al., 2014; Hinduja, 2020).

### 3.2 Hypoperfusion theory

The first working hypothesis after the initial description of PRES by Hinchey et al., in 1996 was that of reduced perfusion (Hinchey et al., 1996), which was based on the assumption that severe systemic arterial hypertension leads to cerebral vasoconstriction, reduced perfusion, ischemia and subsequent vasogenic edema. Reversible cerebral vasoconstriction syndrome (RCVS) is definitely worth mentioning here, as its possible pathophysiological mechanisms and clinical manifestations largely overlap with those of PRES (Bartynski and Boardman, 2008; Singhal et al., 2011; Chen et al., 2010; Cappelen-Smith et al., 2017). Shih-Pin Chen and Shuu-Jiun Wang postulate (Chen and Wang, 2022) that multiple factors such as genetic predisposition (Chen et al., 2011) (including the female sex (Topcuoglu et al., 2016)), medication/drugs (Han et al., 2008; Singhal et al., 2002), childbirth, cold environment (Brown et al., 2003) etc.) trigger endothelial dysfunction (Marra et al., 2014), sympathetic overactivity (Singhal, 2021; Hamel, 2006) and oxidative stress (Faraci, 2006; Yan et al., 2017), which leads to the mechanical (increase in blood pressure, excessive central pulsatile blood flow, shear forces (Chen et al., 2018)) and biochemical (secretion of catecholamines, neuropeptide y, endothelin-1, vasoactive metabolites (Marra et al., 2014), micro-RNA (Chen et al., 2021), proangiogenic factors) phenomena, which in turn cause disruption of the blood-brain barrier (Lee et al., 2017; Pun et al., 2009) and failure of cerebrovascular autoregulation. Above all, endothelial dysfunction (Marra et al., 2014) and disruption of the blood-brain barrier play a decisive role in the development of PRES in patients with sepsis, shock, autoimmune diseases (e.g. lupus, Wegener's granulomatosis), organ transplantation (especially that of allogenic stem cells) and undergoing chemotherapy. It is worth noting that the patients in those subgroups are usually normotensive or even hypotensive. Among the chemotherapeutic agents, ciclosporin and tacrolimus are those frequally mentioned. In those cases, the dysfunction of the endothelium is thought to be caused by inhibition of calcineurin (Holler et al., 1989; Zoja et al., 1986; Wu et al., 2010; Hammerstrom et al., 2013). Marra et al. describe the influence of T-lymphocytes and cytokines (tumor necrosis factor-alpha (TNF-α), interleukin-1 (IL-1), interferon gamma (IFN-γ), Vascular Endothelial Growth Factor (VEGF)) on the function of the endothelium, which leads to a disruption of the blood-brain barrier with resulting edema (Marra et al., 2014). In pregnant women with preeclampsia, a 5-fold increase in plasma VEGF-A levels was detected as compared to the control group (Bills et al., 2011). Last but not least, a reduced perfusion of affected brain areas leads to ischemia (in some cases to the extent of an infarction), which in turn results in cytotoxic edema (Bartynski, 2008b). In case of reperfusion injury or vascular rupture due to severe arterial hypertension, hemorrhage can occur (Doss-Esper et al., 2005).

There is evidence of the sympathetic tone in the area of the anterior circulation to increase the upper limit of autoregulation and to counteract the dilation of the arterioles (Bartynski, 2008b; Hall and Hall, 2021; Roloff et al., 2016). Hence, the predilection of PRES for the brain areas in the posterior circulation is thought to be related to the prevalence of the parasympathetic nervous system (Feske, 2011).

Hinduja et al. postulated an activation of cerebral vasopressin receptors (V1aR), which can also lead to cerebral vasoconstriction, endothelial damage and ischemia with edema formation. This could also result in activation of the renal vasopressin receptors (V2R9) and explain high blood pressure and kidney damage in PRES patients. They therefore assumed that the use of vaptans could play a role in therapy here (Hinduja, 2020).

## 4 Discussion

The case presented here is a female patient with a large VS, who was surgically treated via a retrosigmoid approach. The same constellation was seen in the two known case reports of PRES following such a surgery, where women were treated at a Koos 4 tumor via the same approach. However, the onset of symptoms varies between 2 days and 8 months (Khatri et al., 2018; Sorour et al., 2016). In our case, we found only some of the symptoms, such as somnolence and mild hypertension, which can be associated with PRES (Geocadin, 2023; Gewirtz et al., 2021). However, it should be emphasized again here that these symptoms can have other and much more frequent causes, such as post-hemorrhage, hydrocephalus, meningitis or cerebellar contusion (Dubey et al., 2009; Complications in Neurosurgery, 2019).

The other factor that might have played a role in our case report is the administration of metronidazole. In contrast, metronidazole-induced encephalopathy (MIE) has been frequently described (64 cases in one review), with typically hyperintense lesions of the dentate nucleus of the cerebellum and corpus callosum detected on FLAIR images (Kuriyama et al., 2011). In this literature review, we identified 3 cases of PRES associated with the administration of metronidazole (Gilbert et al., 2020; Hosaka et al., 2023; Kikuchi et al., 2016). The onset of symptoms varies between 1 day, as in our case, and three weeks. Women are more frequently affected (Legriel et al., 2012; Bartynski and Boardman, 2007; Marrone et al., 2016; Datar et al., 2015b) and all the cases we have discussed here in the literature review were female patients. This should be further investigated in terms of pathogenesis, risk factors and prevention options.

The actual reason for administering metronidazole in our case was the presence of CDI. This type of infection after a single course of antibiotic prophylaxis is overall very rare, reported in the literature to be around 1% (Carignan et al., 2008). However, the duration of surgery and repeated administration of antibiotics (over several days) may increase the risk (Branch-Elliman et al., 2019). In our case, due to the duration of surgery of 6 h, the administration of cefazolin was repeated after 4 h, which may well have increased the risk of CDI. With regard to the pathogenesis of PRES, the cytotoxic hypothesis could apply to a PRES resulting from metronidazole. According to Marra et al., endothelial cell damage caused by lactate dehydrogenase (LDH), schistocytes and thrombocytopenia can be seen (Marra et al., 2014). Future investigations may shed light on this.

The extent to which surgery in the posterior cranial fossa can trigger a cytotoxic edema has not yet been described. Sympathetic activation and secondary endothelial damage may arise from local changes in the capillary bed, pressure, local vasospasm, or altered microenvironment. Angiogenesis stimulated by hypoxia with increased permeability or a local immune reaction are also conceivable (Bartynski, 2008b; Chen and Wang, 2022; Bills et al., 2011).

In this presented case, we believe that the administration of metronidazole may have triggered the development of PRES following surgery. In any case, unrecognized cases of PRES may exist after operations with a poor postoperative course and septic symptoms in sedated or comatose ICU patients (Lamy et al., 2014). This case illustrates the complex interplay of factors in the development of PRES. It emphasizes the importance of being vigilant in the treatment of postoperative complications and to consider even atypical causes such as drug-induced PRES.

## 5 Conclusion

The occurrence of PRES after VS resection, although rare, should be recognized as a potential complication. The administration of metronidazole can likewise promote this syndrome. The risk of subsequent CDI was probably increased by the repeated administration of cefazolin during surgery. In this presented case, we assume a mutual intensification of pathophysiological factors through the combination of surgery and metronidazole administration.

The purpose of this review is to raise awareness of a very rare complication such as PRES in this setting. Early diagnosis of this syndrome, prompt intervention and appropriate treatment are crucial to prevent irreversible neurological deficits. Future neuroimaging studies should focus on angiographic imaging and perfusion patterns to characterize cerebral hemodynamics during PRES, which may vary depending on etiologic aspects or disease progression.

## Declaration of competing interest

The authors declare that they have no known competing financial interests or personal relationships that could have appeared to influence the work reported in this paper.
